# Supplementary material for: Agricultural pesticide use and adverse birth outcomes in the San Joaquin Valley of California
Source: Nat Commun. 2017 Aug 29;8:302. doi: 10.1038/s41467-017-00349-2 (PMC5575123; doi:10.1038/s41467-017-00349-2)
Supplement: Supplementary file 1 — Supplementary Information [file 41467_2017_349_MOESM1_ESM.pdf]

File Name: Supplementary Information
Description: Supplementary Figure, Supplementary Tables, Supplementary Note and Supplementary Methods

File Name: Peer Review File
Description:

Supplementary Note 1

Birth Data Details: All data on births came from the California Birth Statistical Master File for 1997-2011, which is the most comprehensive source of birth and demographic data for all births occurring in California. Between 1997-2011 some data items were added, deleted or modified. For the purpose of this study, the three relevant changes are mother's education, tobacco use, and birth abnormalities, all of which were modified in 2006.

Before 2006, mother's education was coded as years of schooling, whereas 2006 and onward, mother's education was binned by degree (e.g. H.S. Degree or equivalent, some college without degree, Associate's, etc.). To make education comparable between these two periods, we recoded mother's education as follows: (1) Less than high school: 0-11 years of schooling (pre-2006); less than high school (2006 onwards), (2) High school degree: 12 years of schooling (pre-2006); high school or equivalent (2006 onwards) (3) Some College: [13-15] years of schooling (pre-2006); some college or associate's degree (2006 onwards) (4) College or more: over 16 years of schooling (pre-2006); Bachelor's, Master's or Professional Degree (2006 onwards); (5) not reported.

Mother's tobacco use also changed during this period. Prior to 2006, tobacco use (binary) was coded as a complication of pregnancy. In 2006, it is missing. Post-2006, data on cigarette use (count per day) is taken by trimester. For our main analysis tables, we exclude 2006 due to the systematic absence of data on the tobacco covariate. Including 2006 without tobacco use as a covariate does not change our main results.

Specific codes within the Abnormal Conditions and Clinical Procedures Relating to the Newborn category also changes. This would cause particular problems for focusing on specific birth abnormalities. We use a binary measure of birth abnormalities as the occurrence of one or

more Abnormal Conditions and Clinical Procedures Relating to the Newborn. Throughout the time period "other" categories remained such as "Additional and unspecified congenital anomalies not listed above" and "Other conditions/procedures not listed", and as such, additions of specific codes such as "Cyanotic congenital heart disease" would not be expected to have a large impact on our presence/absence measure of birth abnormalities.

Finally, some types of birth abnormalities such as heart defects are often missed at birth. Nevertheless, measurement error in the dependent variable will not bias our estimates so long as it is uncorrelated with pesticide exposure.

Supplementary Methods

Since the functional form of the relationship between pesticide use and birth outcomes is unknown and could be non-linear or include exposure threshold effects, we report results from a binary measure of pesticide exposure. Our main analysis focused on a binary split where being in the top 5% of pesticide active ingredients was considered 'high' exposure and below that was considered 'low' exposure. Different binary cutoffs lead to different samples in 'high' and 'low' exposure groups. Thus, for robustness, we tested a 75% and 99%, as well as a continuous model. We found few increases in adverse birth outcomes resulting from being in the 75% of overall exposure or above (~250 kg or above) compared to the low exposure group, with the exception of small increased probability of preterm birth (~5%) for some measures of exposure (Supplementary Table 3). We identified larger coefficients and more significant effects using a 99% cutoff for 'high' exposure for birth weight and low birth weight (maximum ~1% decrease in birth weight and ~20% increase in probability of low birth weight), though less significant effects for gestation and abnormalities (Supplementary Table 3). We also evaluated a binary model comparing the top 5% of exposure to zero exposure in the sample that included all births. We found very similar results to those reported in Table 2A.

In contrast to the binary models, the continuous model assumes a linear relationship between pesticides and birth outcomes, an assumption that would be violated if there were exposure thresholds, saturation effects, or other nonlinearities. For the continuous model we rescaled our measure of exposure to avoid extremely small coefficients. We divided kg of active ingredients by the 95%-5% range of exposure, thus the coefficients represented the change in birth outcomes resulting from being in the 95% of exposure compared to the 5% of exposure. Rescaling continuous variables does not change which coefficients are significant, just the

magnitude due to the change in the ‘units’ of exposure. Nonetheless, we found very small effects for birth weight, low birth weight, gestational length and preterm birth with magnitudes generally much smaller than in the binary 99% or 95% models. Only birth abnormalities were more often found to be statistically significant based on two-sided t-tests of the estimated regression coefficients where the standard errors are clustered at the zipcode level.

To probe the robustness of the results, we also evaluated models with different assumption about clustering adjustment for the standard errors, different location fixed effects, and other environmental variables. Including zipcode-year rather than township-year fixed effects or clustering standard errors at the township rather than zipcode did not change the basic results (Supplementary Tables 4-5). Using data from the California Air Resources Board Ambient Air Quality Monitoring Stations as well as data from the National Climatic Data Center (NCDC) Global Historical Climatology Network-Daily (GHCN-Daily), we included covariates for quantiles of ambient ozone and carbon monoxide concentrations (<70, 70-90, 90-95, >99%), and temperature bins (45, 45-65, 65-85, >85°F; 7.22, 7.22-18.33, 18.33-29.44, >29.44°C) totaled over gestation and by trimester using equivalent methods for attributing trimester and total exposure as we used for pesticides. Daily measures of ambient concentration of pollutants and temperature were assigned to PLS Section using inverse distance weighting from stations within 200 km of the PLS Section centroid. Including these covariates had little effect on our basic results (Supplementary Table 6).

Lastly, using the subsample of observations in Sections with both births and pesticide use in each year, we briefly investigated sex-specific effects of exposure by interacting our exposure measure with sex. Overall there were few significant differences ($\alpha=0.05$) based on two-sided t-tests of the estimated regression coefficients with standard errors clustered at mother’s zipcode.

However, there was some evidence of sex-specific differences in the effect of pesticide exposure on birth weight and gestation, particularly for exposure in the second trimester. Where a difference was observed, males consistently had more negative outcomes. This is consistent with the literature, which suggests male fetuses are more susceptible to environmental exposures. An in-depth analysis of sex-specific differences is outside of the scope of this contribution, but is a valuable research avenue.

Supplementary Table 1. Pesticide Summary Statistics for the Different Samples
Pesticide summary statistics for the entire sample (All Births), for the estimation sample that excluded 2006 due to missing tobacco data (Full Estimation Sample) and for the estimation sample in regions with pesticides and births in each year (Focal Sample). The distribution of pesticides is highly skewed. Binary pesticide cutoffs were based on all births. For all measures of exposure, the focal sample had substantially more pesticide exposure than the full estimation sample. For robustness, we evaluated pesticide exposure calculated in different ways. First, we removed adjuvants (e.g. surfactants), which were not uniformly reported as active ingredients in the Pesticide Use Reports (PUR). Doing so changed total pesticide active ingredients and active ingredients by a very small fraction and did not change our main results (Supplementary Table 7). Second, we calculated trimesters and total gestational exposure from date of last menses forward ('Menses'). Date of last menses was not available for all observations. Analyses based on these fields had sample sizes reduced by 767 and 80 for the full estimation sample and focal sample, respectively. The values correspond to the mean, with standard deviation in parentheses, where applicable.

	All Births	Full Estimation Sample	Focal Sample
Total kg Active Ingredients (AI) during gestation	738 (3989)	748 (4111)	2384 (8328)
Total kg Active Ingredients (AI): 50%	0	0	873
Total kg Active Ingredients (AI): 75%	249	257	2675
Total kg Active Ingredients (AI): 95%	4178	4226	8942
Total kg Active Ingredients (AI): 99%	11134	11213	17842
Total kg AI of toxicity cat. 1 & 2		248 (3589)	882 (7862)
Total kg AI of toxicity cat 3 & 4		333 (1235)	981 (1730)
Total kg AI of aerial pesticides		37 (361)	82 (368)
Total kg AI of ground pesticides		683 (4012)	2195 (8207)
Total kg AI during trimester 1		248 (2144)	797 (4538)
Total kg AI during trimester 2		251 (2165)	797 (4513)
Total kg AI during trimester 3		249 (2266)	790 (4650)
Total kg AI during trimester 0		252 (2531)	811 (5349)
Total kg AI during trimester 4		244 (2631)	794 (5689)
January Birth Month Total kg Active Ingredients		737 (2445)	2355 (4095)
February Birth Month Total kg Active Ingredients		694 (2274)	2209 (3951)
March Birth Month Total kg Active Ingredients		587 (2579)	1919 (5128)
April Birth Month Total kg Active Ingredients		581 (3593)	1930 (7640)
May Birth Month Total kg Active Ingredients		688 (5585)	2297 (12075)
June Birth Month Total kg Active Ingredients		763 (4165)	2381 (8484)
July Birth Month Total kg Active Ingredients		829 (4799)	2595 (9917)
August Birth Month Total kg Active Ingredients		874 (4489)	2686 (8527)
September Birth Month Total kg Active Ingredients		850 (4421)	2704 (8651)
October Birth Month Total kg Active Ingredients		826 (4973)	2627 (10359)
November Birth Month Total kg Active Ingredients		755 (4189)	2383 (8168)
December Birth Month Total kg Active Ingredients		752 (3990)	2389 (8026)
Within Section Standard Deviation: Total kg		1672	2018
Within Section Standard Deviation: Trimester 1		961	1297
Within Section Standard Deviation: Trimester 2		994	1220
Within Section Standard Deviation: Trimester 3		1016	1251
Within Section Standard Deviation: Tox. cat. 1 & 2		638	952
Within Section Standard Deviation: Tox. cat. 3 & 4		1029	1018
Robustness			
Total kg AI during gestation (No Adjuvants)		743 (4105)	2367 (8320)
Total kg AI during trimester 1 (No Adjuvants)		246 (2142)	791 (4536)
Total kg AI during trimester 2 (No Adjuvants)		249 (2164)	792 (4511)
Total kg AI during trimester 3 (No Adjuvants)		247 (2265)	785 (4647)
Total kg AI during gestation (Menses)		751 (4209)*	2393 (8561)*
Total kg AI during trimester 1 (Menses)		249 (2260)*	802 (4795)*
Total kg AI during trimester 2 (Menses)		253 (2331)*	807 (4912)*
Total kg AI during trimester 3 (Menses)		248 (2154)*	784 (4375)*
N	753290	692586	137210

Supplementary Table 2. Covariates for the Model of Log Birth Weight and Low Birth Weight in the Focal Sample
Covariates corresponding to the model in Table 2B for low birth weight and log birth weight. For all regression tables except Supplementary Table 5, statistical significance is based on two-tailed t-tests of the estimated regression coefficient with standard errors clustered at mother's zipcode. For all regression tables, covariates are above the standard errors in parentheses, and statistical significance is indicated by * for p<0.05 and ** for p<0.01. Bold indicates statistical significance of the pesticide exposure covariates in the expected direction.

	(1)	(2)	(3)	(4)	(5)	(6)
		Log Birth weight			Low Birth Weight	
Pesticide Exposure						
Total AI	**-0.00387***			0.00213		
	**(0.00162)**			(0.00192)		
Trimester 1		**-0.00362***			0.00146	
		**(0.00149)**			(0.00142)	
Trimester 2		-0.00194			**0.00363***	
		(0.00132)			**(0.00147)**	
Trimester 3		0.000725			-0.000161	
		(0.00153)			(0.00176)	
"High" acute tox.			-0.00152			0.00224
			(0.00169)			(0.00208)
"Low" acute tox.			-0.000235			-0.00275
			(0.00182)			(0.00182)
Mother's Age						
(<19 = reference)						
19-24	0.0113**	0.0113**	0.0113**	-0.00488*	-0.00489*	-0.00489*
	(0.00182)	(0.00183)	(0.00182)	(0.00235)	(0.00236)	(0.00235)
25-35	0.0190**	0.0190**	0.0190**	-0.00101	-0.000991	-0.00101
	(0.00181)	(0.00181)	(0.00181)	(0.00253)	(0.00253)	(0.00253)
35 +	0.0130**	0.0130**	0.0130**	0.0178**	0.0178**	0.0178**
	(0.00301)	(0.00301)	(0.00301)	(0.00337)	(0.00338)	(0.00337)
Infant Sex						
Female	-0.0295**	-0.0295**	-0.0295**	0.00271*	0.00272*	0.00271*
	(0.000915)	(0.000913)	(0.000914)	(0.00118)	(0.00118)	(0.00118)
Unreported	-0.714*	-0.715*	-0.714*	0.448	0.448	0.448
	(0.288)	(0.288)	(0.288)	(0.246)	(0.246)	(0.246)
Mother's Education						
(<H.S. = reference)						

H.S.	-0.00285*	-0.00285*	-0.00285*	0.00243	0.00243	0.00244
	(0.00133)	(0.00134)	(0.00134)	(0.00143)	(0.00143)	(0.00143)
Some college	-0.00132	-0.00130	-0.00130	0.00391*	0.00392*	0.00392*
	(0.00161)	(0.00161)	(0.00161)	(0.00162)	(0.00162)	(0.00163)
College+	0.00716**	0.00719**	0.00718**	-0.00587**	-0.00586**	-0.00583*
	(0.00212)	(0.00213)	(0.00213)	(0.00224)	(0.00224)	(0.00224)
Missing	-0.000434	-0.000411	-0.000369	0.0104	0.0104	0.0103
	(0.00689)	(0.00690)	(0.00691)	(0.00789)	(0.00789)	(0.00789)
Mother's Race-Ethnicity (White-non-Hispanic =reference)						
Non-Hispanic Black	-0.0620**	-0.0620**	-0.0620**	0.0346**	0.0346**	0.0345**
	(0.00538)	(0.00537)	(0.00538)	(0.00668)	(0.00668)	(0.00667)
Hispanic	-0.00662**	-0.00664**	-0.00665**	-0.00122	-0.00121	-0.00122
	(0.00163)	(0.00163)	(0.00164)	(0.00162)	(0.00162)	(0.00162)
Other Race	-0.0472**	-0.0472**	-0.0472**	0.0136**	0.0136**	0.0136**
	(0.00296)	(0.00295)	(0.00296)	(0.00337)	(0.00337)	(0.00337)
Race missing	-0.00905	-0.00896	-0.00905	0.00326	0.00318	0.00325
	(0.00975)	(0.00976)	(0.00976)	(0.0108)	(0.0108)	(0.0108)
Pre-Natal Care (<5 =reference)						
5-8 visits	0.0289**	0.0289**	0.0289**	-0.0202**	-0.0201**	-0.0202**
	(0.00414)	(0.00415)	(0.00414)	(0.00449)	(0.00450)	(0.00449)
9-14 visits	0.0775**	0.0775**	0.0775**	-0.0689**	-0.0689**	-0.0689**
	(0.00410)	(0.00410)	(0.00410)	(0.00429)	(0.00429)	(0.00429)
15+ visits	0.0803**	0.0803**	0.0803**	-0.0695**	-0.0694**	-0.0694**
	(0.00460)	(0.00459)	(0.00460)	(0.00462)	(0.00461)	(0.00461)
Missing	0.0500**	0.0500**	0.0500**	-0.0366**	-0.0366**	-0.0366**
	(0.00762)	(0.00762)	(0.00763)	(0.00832)	(0.00832)	(0.00833)
Other Covariates						
Tobacco Use	-0.0504**	-0.0504**	-0.0504**	0.0353**	0.0354**	0.0353**
	(0.00521)	(0.00521)	(0.00521)	(0.00699)	(0.00699)	(0.00699)
First child	-0.0311**	-0.0311**	-0.0311**	0.0213**	0.0213**	0.0213**
	(0.00147)	(0.00146)	(0.00147)	(0.00148)	(0.00148)	(0.00149)
Month of Birth (January =reference)						
February	9.03e-05	-3.28e-05	0.000141	0.000254	0.000340	0.000181
	(0.00227)	(0.00229)	(0.00227)	(0.00239)	(0.00239)	(0.00239)
March	-0.00145	-0.00172	-0.00130	-0.000120	-3.71e-05	-0.000383
	(0.00240)	(0.00241)	(0.00243)	(0.00276)	(0.00277)	(0.00279)
April	-7.24e-05	-0.000439	8.15e-05	0.00191	0.00192	0.00162

	(0.00218)	(0.00221)	(0.00220)	(0.00270)	(0.00272)	(0.00271)
May	-0.000231	-0.000673	-0.000148	-0.000853	-0.000930	-0.00100
	(0.00238)	(0.00240)	(0.00239)	(0.00254)	(0.00256)	(0.00256)
June	-0.00248	-0.00299	-0.00249	0.00303	0.00299	0.00304
	(0.00232)	(0.00237)	(0.00233)	(0.00279)	(0.00283)	(0.00279)
July	5.26e-05	-0.000366	-9.50e-06	-0.000167	-0.000206	-5.11e-05
	(0.00213)	(0.00215)	(0.00212)	(0.00288)	(0.00291)	(0.00287)
August	-0.00110	-0.00134	-0.00118	-0.000962	-0.00114	-0.000794
	(0.00211)	(0.00217)	(0.00211)	(0.00264)	(0.00263)	(0.00263)
September	-0.00146	-0.00155	-0.00154	-0.000828	-0.00117	-0.000629
	(0.00253)	(0.00259)	(0.00254)	(0.00294)	(0.00296)	(0.00295)
October	-0.00389	-0.00388	-0.00392	0.000960	0.000542	0.00106
	(0.00227)	(0.00228)	(0.00227)	(0.00264)	(0.00264)	(0.00264)
November	-0.00138	-0.00130	-0.00139	-0.00108	-0.00142	-0.00104
	(0.00247)	(0.00249)	(0.00248)	(0.00294)	(0.00296)	(0.00294)
December	-0.00407	-0.00401	-0.00406	0.000625	0.000493	0.000624
	(0.00227)	(0.00228)	(0.00227)	(0.00311)	(0.00314)	(0.00311)
Constant	8.065**	8.065**	8.064**	0.0936**	0.0932**	0.0940**
	(0.00443)	(0.00445)	(0.00450)	(0.00549)	(0.00554)	(0.00558)
Observations	137210	137210	137210	137210	137210	137210

Supplementary Table 3. Different Specification of Pesticide Exposure on Birth Outcomes Binary pesticide exposure at different cutoffs for high exposure (>75%; A) or (>99%; B), and a continuous specification (C) with kg of active ingredients scaled by the 95-5% range. Total AI indicates total kg of pesticide active ingredients applied in the 2.6 km^2 region encompassing mothers addresses, 'Tox' indicates total kg of active ingredients by toxicity categories based on the EPA signal word, and trimester indicates total kg by trimester. The focal sample is observations in 2.6 km^2 regions that have births and pesticides in all years (corresponding to Table 2B; "focal sample").

	Log BW	Low BW	Log gestation	Preterm	Any Abnormalities
**A. Binary, 75%=1**					
Total AI	0.00138<br>(0.00148)	-0.00307<br>(0.00158)	-0.000706<br>(0.000400)	0.00370<br>(0.00224)	-0.00329<br>(0.00190)
Tox Cat 1 & 2	-0.00105<br>(0.00166)	0.00262<br>(0.00149)	0.000124<br>(0.000508)	-0.000735<br>(0.00226)	-0.000291<br>(0.00225)
Tox Cat 3 & 4	4.36e-05<br>(0.00149)	-0.000154<br>(0.00169)	-0.000565<br>(0.000483)	**0.00514***<br>**(0.00228)**	-8.71e-05<br>(0.00173)
Trimester 1	0.000325<br>(0.00143)	0.000901<br>(0.00168)	-0.000430<br>(0.000468)	0.00233<br>(0.00208)	-1.15e-05<br>(0.00159)
Trimester 2	-0.000466<br>(0.00125)	**-0.00314***<br>**(0.00121)**	0.000248<br>(0.000458)	-0.00224<br>(0.00193)	-0.000873<br>(0.00150)
Trimester 3	-0.000698<br>(0.00140)	0.000904<br>(0.00139)	-0.000663<br>(0.000403)	**0.00458***<br>**(0.00195)**	-0.000738<br>(0.00189)
	N=137210	N=137210	N=137210	N=137210	N=136621
**B. Binary, 99%=1**					
Total AI	-0.00653<br>(0.00347)	**0.00914****<br>**(0.00333)**	0.000161<br>(0.000982)	-0.000382<br>(0.00507)	0.00332<br>(0.00431)
Tox Cat 1 & 2	**-0.00999****<br>**(0.00287)**	**0.00848***<br>**(0.00383)**	-0.000658<br>(0.00102)	0.00389<br>(0.00410)	0.000691<br>(0.00522)
Tox Cat 3 & 4	**-0.00840***<br>**(0.00339)**	**0.00682***<br>**(0.00321)**	-0.00230<br>(0.00125)	0.00399<br>(0.00573)	0.000831<br>(0.00411)
Trimester 1	-0.00522<br>(0.00369)	0.00148<br>(0.00323)	2.07e-06<br>(0.00105)	-0.00175<br>(0.00569)	-0.00127<br>(0.00438)
Trimester 2	**-0.00549***<br>**(0.00223)**	0.00467<br>(0.00267)	-0.00163<br>(0.000886)	**0.0109***<br>**(0.00512)**	0.00455<br>(0.00461)
Trimester 3	-0.00233<br>(0.00316)	0.00440<br>(0.00375)	-0.00152<br>(0.000933)	0.00716<br>(0.00442)	0.00306<br>(0.00427)
	N=137210	N=137210	N=137210	N=137210	N=136621
**C. Continuous, scaled by 95%-5% range**					
Total AI	-0.000267<br>(0.000225)	0.000470<br>(0.000264)	**-0.000129****<br>**(4.74e-05)**	**0.000599****<br>**(0.000214)**	**0.00144****<br>**(0.000122)**
Tox Cat 1 & 2	-4.71e-05<br>(4.18e-05)	8.46e-05<br>(5.00e-05)	**-1.57e-05****<br>**(3.18e-06)**	**7.50e-05****<br>**(2.03e-05)**	**0.000274****<br>**(1.99e-05)**
Tox Cat 3 & 4	-0.00134<br>(0.000802)	0.000762<br>(0.000627)	**-0.000862****<br>**(0.000199)**	**0.00302****<br>**(0.00101)**	0.00109<br>(0.000849)
Trimester 1	-7.12e-05<br>(8.29e-05)	5.63e-05<br>(5.67e-05)	2.39e-05<br>(2.23e-05)	4.57e-05<br>(0.000101)	**0.000296****<br>**(4.66e-05)**
Trimester 2	-2.62e-05<br>(0.000108)	**0.000245***<br>**(0.000111)**	**-8.53e-05****<br>**(2.30e-05)**	**0.000473****<br>**(0.000105)**	**0.000756****<br>**(4.63e-05)**
Trimester 3	**-0.000133****<br>**(5.08e-05)**	0.000115<br>(0.000105)	**-5.17e-05****<br>**(1.89e-05)**	2.19e-05<br>(9.08e-05)	**0.000235****<br>**(6.38e-05)**
	N=137210	N=137210	N=137210	N=137210	N=136621

Supplementary Table 4. Different Fixed Effects Model Specifications
Including zipcode * year and month fixed effects (B), or PLS Township, year and month fixed effects (C) did not change the overall conclusions of the original analysis (A).

	Log BW	Low BW	Log gestation	Preterm	Any Abnormalities
**A. Original, Township x Year + Month FE**					
Total AI	**-0.00387*** **(0.00162)**	0.00213 (0.00192)	**-0.00132*** **(0.000558)**	**0.00745**** **(0.00275)**	**0.00504*** **(0.00225)**
Tox Cat 1 & 2	-0.00152 (0.00169)	0.00224 (0.00208)	-0.000273 (0.000504)	0.00318 (0.00264)	0.00128 (0.00193)
Tox Cat 3 & 4	-0.000235 (0.00182)	-0.00275 (0.00182)	**-0.00135**** **(0.000492)**	0.00432 (0.00248)	0.00249 (0.00236)
Trimester 1	**-0.00362*** **(0.00149)**	0.00146 (0.00142)	-0.000476 (0.000548)	0.00276 (0.00289)	0.00163 (0.00175)
Trimester 2	-0.00194 (0.00132)	**0.00363*** **(0.00147)**	-0.000647 (0.000442)	**0.00498*** **(0.00221)**	0.00247 (0.00189)
Trimester 3	0.000725 (0.00153)	-0.000161 (0.00176)	-0.000401 (0.000532)	0.000780 (0.00253)	-0.000917 (0.00204)
	N=137210	N=137210	N=137210	N=137210	N=136621
**B. Zip x Year + Month FE**					
Total AI	-0.00252 (0.00154)	0.00146 (0.00165)	**-0.00111*** **(0.000539)**	**0.00596*** **(0.00279)**	0.00372 (0.00219)
Tox Cat 1 & 2	7.14e-05 (0.00149)	0.00121 (0.00188)	-0.000112 (0.000526)	0.00306 (0.00254)	0.000936 (0.00223)
Tox Cat 3 & 4	-0.00121 (0.00173)	-0.00127 (0.00198)	**-0.00127*** **(0.000529)**	0.00448 (0.00268)	0.00148 (0.00241)
Trimester 1	**-0.00313*** **(0.00141)**	0.000864 (0.00150)	-0.000208 (0.000572)	0.00149 (0.00291)	0.000855 (0.00174)
Trimester 2	-0.00172 (0.00137)	**0.00370*** **(0.00148)**	-0.000486 (0.000457)	0.00433 (0.00231)	0.00188 (0.00186)
Trimester 3	0.00152 (0.00147)	-0.000477 (0.00184)	-0.000274 (0.000524)	0.000187 (0.00246)	-0.00150 (0.00205)
	N=137210	N=137210	N=137210	N=137210	N=136621
**C. Township + Year + Month FE**					
Total AI	**-0.00324*** **(0.00150)**	0.00278 (0.00177)	-0.000880 (0.000517)	**0.00506*** **(0.00252)**	0.00311 (0.00256)
Tox Cat 1 & 2	-0.00149 (0.00151)	0.00225 (0.00188)	-0.000115 (0.000403)	0.00309 (0.00218)	-0.000278 (0.00207)
Tox Cat 3 & 4	-0.00100 (0.00165)	-0.00140 (0.00178)	**-0.00141**** **(0.000465)**	**0.00480*** **(0.00240)**	0.00352 (0.00209)
Trimester 1	**-0.00364*** **(0.00143)**	0.00175 (0.00146)	-0.000356 (0.000537)	0.00150 (0.00274)	0.000472 (0.00188)
Trimester 2	-0.00184 (0.00137)	**0.00412**** **(0.00144)**	-0.000479 (0.000478)	0.00387 (0.00227)	0.00132 (0.00174)
Trimester 3	0.000979 (0.00155)	4.64e-05 (0.00172)	-0.000298 (0.000538)	-9.09e-05 (0.00253)	-0.00208 (0.00208)
	N=137210	N=137210	N=137210	N=137210	N=136621

Supplementary Table 5. Different Standard Error Specifications Using the Focal Sample
Clustering the standard errors at the Township of the mother's address (B) rather than the zipcode (A) did not change the overall patterns observed. Statistical significance is based on two-tailed t-tests of the estimated regression coefficient with standard errors clustered at mother's zipcode (A) and at the mother's Township (B).

	Log BW	Low BW	Log gestation	Preterm	Any Abnormalities
**A. Original Zipcode SE**					
Total AI	**-0.00387*<br>(0.00162)**	0.00213<br>(0.00192)	**-0.00132*<br>(0.000558)**	**0.00745**<br>(0.00275)**	**0.00504*<br>(0.00225)**
Tox Cat 1 & 2	-0.00152<br>(0.00169)	0.00224<br>(0.00208)	-0.000273<br>(0.000504)	0.00318<br>(0.00264)	0.00128<br>(0.00193)
Tox Cat 3 & 4	-0.000235<br>(0.00182)	-0.00275<br>(0.00182)	**-0.00135**<br>(0.000492)**	0.00432<br>(0.00248)	0.00249<br>(0.00236)
Trimester 1	**-0.00362*<br>(0.00149)**	0.00146<br>(0.00142)	-0.000476<br>(0.000548)	0.00276<br>(0.00289)	0.00163<br>(0.00175)
Trimester 2	-0.00194<br>(0.00132)	**0.00363*<br>(0.00147)**	-0.000647<br>(0.000442)	**0.00498*<br>(0.00221)**	0.00247<br>(0.00189)
Trimester 3	0.000725<br>(0.00153)	-0.000161<br>(0.00176)	-0.000401<br>(0.000532)	0.000780<br>(0.00253)	-0.000917<br>(0.00204)
	N=137210	N=137210	N=137210	N=137210	N=136621
**A. Township SE**					
Total AI	**-0.00387*<br>(0.00172)**	0.00213<br>(0.00200)	**-0.00132*<br>(0.000556)**	**0.00745**<br>(0.00256)**	0.00504<br>(0.00289)
Tox Cat 1 & 2	-0.00152<br>(0.00207)	0.00224<br>(0.00217)	-0.000273<br>(0.000556)	0.00318<br>(0.00265)	0.00128<br>(0.00182)
Tox Cat 3 & 4	-0.000235<br>(0.00193)	-0.00275<br>(0.00184)	**-0.00135**<br>(0.000506)**	0.00432<br>(0.00238)	0.00249<br>(0.00272)
Trimester 1	**-0.00362*<br>(0.00163)**	0.00146<br>(0.00150)	-0.000476<br>(0.000539)	0.00276<br>(0.00241)	0.00163<br>(0.00154)
Trimester 2	-0.00194<br>(0.00136)	**0.00363*<br>(0.00161)**	-0.000647<br>(0.000482)	**0.00498*<br>(0.00197)**	0.00247<br>(0.00188)
Trimester 3	0.000725<br>(0.00125)	-0.000161<br>(0.00168)	-0.000401<br>(0.000543)	0.000780<br>(0.00266)	-0.000917<br>(0.00176)
	N=137210	N=137210	N=137210	N=137210	N=136621

Supplementary Table 6. Specifications Including Covariates for Other Environmental Factors Including a rich set of covariates for ozone, carbon monoxide and temperature corresponding to total and by trimester exposure (B), did little to modify the observed effects of pesticides over gestation or by trimester (A). Ozone & CO bins are quantiles, <70, 70-90, 90-95, >99%, and temperature bins are 45, 45-65, 65-85, >85°F; 7.22, 7.22-18.33, 18.33-29.44, >29.44°C.

	Log BW	Low BW	Log gestation	Preterm	Any Abnormalities
A. **No Pollution or Temp.**					
Total AI	**-0.00387*<br>(0.00162)**	0.00213<br>(0.00192)	**-0.00132*<br>(0.000558)**	**0.00745**<br>(0.00275)**	**0.00504*<br>(0.00225)**
Trimester 1	**-0.00362*<br>(0.00149)**	0.00146<br>(0.00142)	-0.000476<br>(0.000548)	0.00276<br>(0.00289)	0.00163<br>(0.00175)
Trimester 2	-0.00194<br>(0.00132)	**0.00363*<br>(0.00147)**	-0.000647<br>(0.000442)	**0.00498*<br>(0.00221)**	0.00247<br>(0.00189)
Trimester 3	0.000725<br>(0.00153)	-0.000161<br>(0.00176)	-0.000401<br>(0.000532)	0.000780<br>(0.00253)	-0.000917<br>(0.00204)
	N=137210	N=137210	N=137210	N=137210	N=136621
**B. Ozone, CO & Temp.**					
Total AI	**-0.00405*<br>(0.00163)**	0.00221<br>(0.00198)	**-0.00132*<br>(0.000582)**	**0.00777**<br>(0.00288)**	**0.00502*<br>(0.00228)**
Trimester 1	**-0.00343*<br>(0.00155)**	0.00141<br>(0.00144)	-0.000500<br>(0.000538)	0.00237<br>(0.00281)	0.00185<br>(0.00184)
Trimester 2	-0.00197<br>(0.00140)	**0.00341*<br>(0.00152)**	-0.000604<br>(0.000461)	**0.00507*<br>(0.00216)**	0.00233<br>(0.00188)
Trimester 3	0.000497<br>(0.00151)	3.78e-05<br>(0.00178)	-0.000408<br>(0.000547)	0.00118<br>(0.00249)	-0.000438<br>(0.00202)
	N=134071	N=134071	N=1134071	N=1134071	N=133494

Supplementary Table 7. Different Measure of Gestational Length and Pesticide Exposure
The effect of pesticide exposure on birth outcomes for births with gestation <44wks (B), removing adjuvants from pesticide totals (C), gestation less than 44 weeks, no adjuvants, and calculating trimester (and thus total) from date of last menses forward (D) did not change overall patterns of the magnitude and direction of effects of pesticide exposure on adverse birth outcomes, though which coefficients were significant did change in limited instances relative to the original model (A).

	Log BW	Low BW	Log gestation	Preterm	Any Abnormalities
**A. Original**					
Sum AI	**-0.00387* (0.00162)**	0.00213 (0.00192)	**-0.00132* (0.000558)**	**0.00745** (0.00275)**	**0.00504* (0.00225)**
Trimester 1	**-0.00362* (0.00149)**	0.00146 (0.00142)	-0.000476 (0.000548)	0.00276 (0.00289)	0.00163 (0.00175)
Trimester 2	-0.00194 (0.00132)	**0.00363* (0.00147)**	-0.000647 (0.000442)	**0.00498* (0.00221)**	0.00247 (0.00189)
Trimester 3	0.000725 (0.00153)	-0.000161 (0.00176)	-0.000401 (0.000532)	0.000780 (0.00253)	-0.000917 (0.00204)
N	137210	137210	137210	137210	136621
**B. Gestation < 44wks**					
Sum AI	**-0.00443* (0.00171)**	0.00252 (0.00202)	**-0.00145** (0.00055)**	**0.00767** (0.00283)**	**0.00516* (0.00239)**
Trimester 1	**-0.00394* (0.00153)**	0.00180 (0.00147)	-0.00078 (0.00049)	0.00300 (0.00295)	0.00165 (0.00181)
Trimester 2	-0.00219 (0.00137)	**0.00377* (0.00149)**	-0.00077 (0.00043)	**0.00517* (0.00227)**	0.00271 (0.00194)
Trimester 3	0.000424 (0.00155)	-0.00009 (0.00182)	-0.00054 (0.00048)	0.00085 (0.00257)	-0.00108 (0.00210)
N	133,690	133,690	133,690	133,690	133,118
**C. No Adjuvants**					
Total AI	**-0.00406* (0.00156)**	0.00213 (0.00190)	**-0.00137* (0.00056)**	**0.00720** (0.00274)**	**0.00551* (0.00230)**
Trimester 1	**-0.00348* (0.00155)**	0.00119 (0.00147)	-0.00042 (0.00057)	0.00247 (0.00297)	0.00156 (0.00178)
Trimester 2	-0.00196 (0.00132)	**0.00351* (0.00153)**	-0.00072 (0.00044)	**0.00513* (0.00220)**	0.00279 (0.00195)
Trimester 3	0.00070 (0.00155)	-0.00011 (0.00176)	-0.00031 (0.00054)	0.00020 (0.00252)	-0.00121 (0.00208)
N	137210	137210	137210	137210	136621
**D. Gestation<44wks, No adjuvants, Gestation determined from menses,**					
Total AI	-0.00226 (0.00171)	0.00163 (0.00222)	**-0.00132** (0.000501)**	**0.00756** (0.00243)**	**0.00488* (0.00225)**
Trimester 1	**-0.00332* (0.00137)**	0.00288 (0.00160)	-0.00065 (0.00050)	0.00401 (0.00265)	0.00262 (0.00187)
Trimester 2	**-0.00307* (0.00121)**	**0.00382* (0.00155)**	-0.00096 (0.00049)	0.00395 (0.00260)	**0.00353* (0.00176)**
Trimester 3	0.00263 (0.00171)	*-0.00365* (0.00170)*	0.00066 (0.00044)	-0.00184 (0.00254)	-0.00348 (0.00214)
N	133,632	133,632	133,632	133,632	133061

Supplementary Table 8. List of Birth Abnormalities and Their Rate Per 100,000 Births

Years	Code	Description	Rate per 100k births
97-11	73	NICU admission	3681.6
97-11	75	Other conditions/procedures not listed	2316.4
97-11	74	Newborn transferred to another facility within 24 hours of delivery	727.1
97-11	71	Assisted ventilation required immediately following delivery	433.6
06-11	87	Antibiotics received by the newborn for suspected neonatal sepsis	274.6
97-11	66	Significant birth injury (skeletal fractures, peripheral nerve injury, soft tissue or solid organ hemorrhage which requires intervention)	130.9
06-11	85	Assisted ventilation required for more than 6 hours	94.5
97-11	62	Additional and unspecified congenital anomalies (not listed)	35.8
97-11	30	Cleft palate with cleft lip	22.8
06-11	79	Gastroschisis	22.3
97-11	57	Down’s syndrome (karyotype confirmed)	17
97-11	29	Cleft lip alone	15.1
06-11	86	Newborn given surfactant replacement therapy	14.5
06-11	81	Down’s syndrome (karyotype pending)	13.3
97-11	28	Cleft palate alone	8.9
97-11	35	Hypospadias	8.6
06-11	83	Suspected chromosomal disorder (karyotype pending)	7.2
97-11	1	Anencephaly	6.8
97-11	2	Meningiomyelocele/Spina bifida	6.8
06-11	76	Cyanotic congenital heart disease	6.6
97-11	70	Seizure or serious neurological dysfunction	6.2
06-11	80	Limb reduction defect (excluding congenital amputation and dwarfing syndromes)	5.7
06-11	77	Congenital diaphragmatic hernia	3.3
06-11	82	Suspected chromosomal disorder (karyotype confirmed)	2.7
06-11	88	Aortic stenosis	1.5
06-11	90	Atresia	1.5
06-11	78	Omphalocele	1.2
06-11	89	Pulmonary stenosis	1.2

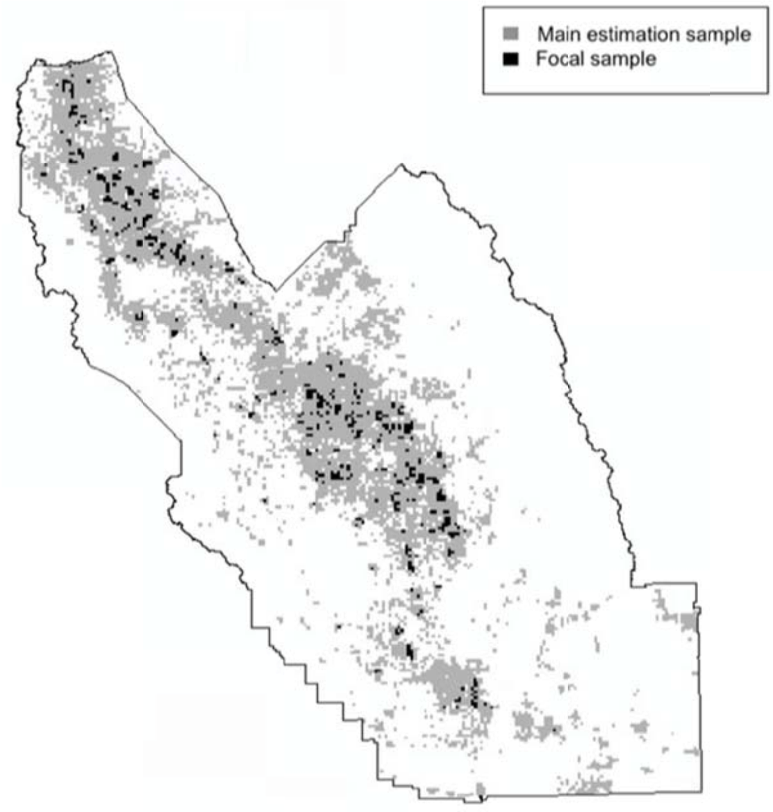

Supplementary Fig 1. Geographic Distribution of Different Samples
The main estimation sample (gray) and focal sample (black) within the San Joaquin Valley are mapped at the Public Land Survey Section ($2.6km^2$) level.
